# Vertical serpentine interconnect-enabled stretchable and curved electronics

**DOI:** 10.1038/s41378-023-00625-w

**Published:** 2023-11-27

**Authors:** Rui Jiao, Ruoqin Wang, Yixin Wang, Yik Kin Cheung, Xingru Chen, Xiaoyi Wang, Yang Deng, Hongyu Yu

**Affiliations:** 1 Department of Mechanical and Aerospace Engineering, The Hong Kong University of Science and Technology, Kowloon, Hong Kong SAR, 999077 China; 2 HKUST Shenzhen-Hong Kong Collaborative Innovation Research Institute, Shenzhen, Guangdong 518045 China

**Keywords:** Electrical and electronic engineering, Structural properties

## Abstract

Stretchable and curved electronic devices are a promising technology trend due to their remarkable advantages. Many approaches have been developed to manufacture stretchable and curved electronics. Here, to allow such electronics to better serve practical applications, ranging from wearable devices to soft robotics, we propose a novel vertical serpentine conductor (VSC) with superior electrical stability to interconnect functional devices through a silicon-based microfabrication process. Conformal vacuum transfer printing (CVTP) technology was developed to transfer the networked platform onto complex curved surfaces to demonstrate feasibility. The mechanical and electrical performance were investigated numerically and experimentally. The VSC interconnected network provides a new approach for stretchable and curved electronics with high stretchability and reliability.

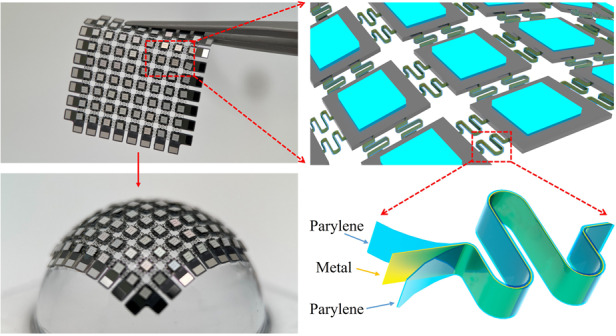

## Introduction

Recently, curved electronics have developed rapidly due to the omnipresent nature of curved surfaces. Compared with traditional electronic devices, which are inherently rigid and fragile, curved electronics can be stretched and bent to conform to the desired surface curvature while maintaining their electrical properties. Based on these unique advantages, curved electronic technologies have great potential for electronic devices in various application scenarios, including wearable devices^1–3^, smart contact lenses^4–6^, soft robotics^7–9^, smart skins^10^, flexible displays^11^, and energy harvesting^12–14^.

Several technologies for manufacturing curved electronics have recently emerged, including the direct fabrication of materials onto curved surfaces and the indirect transformation of planar-manufactured deformable devices into curved shapes. The former approach utilizes technologies such as inkjet printing^15,16^, 3D printing^17,18^, and laser direct writing^19–21^ to directly construct curved electronics. While these methods provide a cost-effective approach to manufacture curved devices, they have limitations in printed materials and structures, and the electrical performances are also far inferior to the devices manufactured using standard 2D fabrication technology. The latter strategy is to manufacture devices in a planar form and then transform them into desired curved shapes, which results in higher performance and more versatility in application. In this approach, stretchability is crucial to enhance the compatibility level and allow devices to conform to complex curved surfaces, particularly those with nonzero Gaussian curvatures^22^, such as spherical and saddle surfaces.

Generally, stretchability can be obtained through two approaches: using intrinsically stretchable materials or structure-enabled stretchability. The first strategy often involves elastic polymeric substrates, such as polydimethylsiloxane, hydrogels, and Ecoflex, along with conductive fillers such as carbon nanotubes, metal nanowires, and graphene to create stretchable and conductive composites^23–25^. Although such composites have advantages including natural flexibility, stretchability and low cost, the fatal drawback is that the conductivity is far lower than that of conventional conductors. The second strategy utilizes structural designs to assist conventional materials in achieving device-level stretchability. For example, a prestretched PDMS substrate was utilized to form a wavy buckled structure^26,27^, which was repeatedly stretched and compressed without damaging thin films. Following that, many generations of island-bridge designs^28^ were proposed to obtain high stretchability. The islands are rigid and mounted functional components interconnected by stretchable bridges with special structural designs, such as arc-shaped structures^29,30^, in-plane serpentine structures^31–34^, and 3D helical forms^35,36^. The formation of arc-shaped structures is similar to the wavy buckled structure, resulting in limited stretchability. The island-bridge design using the in-plane serpentine structure can realize high-level stretchability. However, in-plane serpentine interconnects often have sharp stress concentration problems during stretching deformation^37^. Optimizing the geometric dimensions of serpentine strips^38,39^ can improve this issue but results in a trade-off between the mechanical properties and electrical performance. Recently, several fabrication methods have been developed to obtain serpentine structures with high aspect ratios^40,41^, but coupling with PDMS brings application limitations and functional degradation during long-term usage. 3D helical interconnects^42,43^ formed by a prestretched elastomer substrate can also relieve stress concentrations through uniform out-of-plane deformations. One of the challenges, however, is their incompatibility with mature industrial manufacturing processes due to the use of elastomeric substrates in the core step, which hinders future mass production.

After manufacturing deformable devices, transformation is needed to obtain curved electronics^44–46^. Several technologies have been developed to assist the transformation process, but all suffer from some deficiencies. For example, prestretching and releasing elastomeric membranes with curved shapes were proposed to pick up and transfer electronic devices in 2D geometry to 3D forms^4^. Unfortunately, the prestretching of the elastomer membrane limits it to simple curved shapes and low area usage. Elastomer materials such as polydimethylsiloxane (PDMS) and Eco-flex always suffer from fatigue problems, which influence the normal usage of the device. Another approach adopted a conformal additive stamp printing method during the transfer process, where a pneumatically inflated elastomeric balloon is used to pick up and print 2D devices^46^. This approach can be applied to various applications and arbitrary curved shapes. However, location distortion occurs due to multiple transformations between planar and curved forms. Notably, origami tessellation can also be applied to achieve stretchable and curved electronics^47–50^ as a structure-enabled curved device. Deng and coworkers^48^ utilized a designed and optimized 2D origami tessellation manufactured in wafer-based technology. After peeling off from the silicon wafer, the tessellation was converted into a three-dimensional curved structure along the zigzag crease. The origami folding endows the electronic device with system-level stretchability, allowing the device to wrap closely onto predesigned curved surfaces. However, devices mounted on each origami unit are angled after folding, resulting in undesirable properties, and the substrate also limits its application scenarios.

**Fig. 1:**
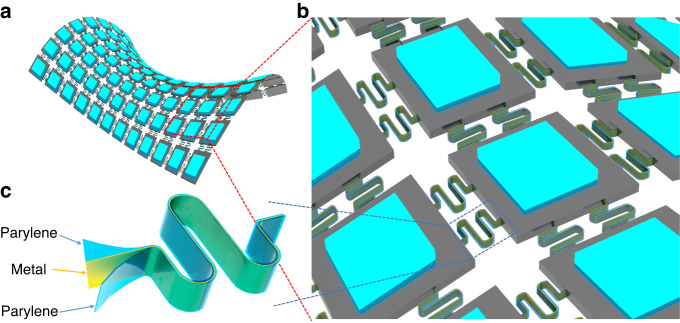
Schematic diagram of VSC-enabled curved electronics. **a** Curved electronics interconnected with vertical serpentine conductors. **b** Magnified pictures of curved electronics in (**a**). **c** Schematic diagram of the vertical serpentine conductor, including three layers, where the metal layer is arranged near the mechanical neutral plane

Although various technologies have been developed to improve the conformability of curved electronic devices when integrated onto arbitrarily curved surfaces, challenges still exist. In this report, we combine silicon-based MEMS fabrication technology and a transfer printing strategy to manufacture curved electronic devices. A vertical serpentine conductor (VSC) was proposed and manufactured to solve the sharp stress concentrations at arc regions during the extension and contraction of interconnects and improve the conformability and reliability of island-bridge constructed curved electronics (Fig. 1). The metal layer was arranged near the mechanical neutral plane by adjusting the thickness of the bottom Parylene-C layer to improve the mechanical properties of the composite structure for interconnects (Fig. 1c). The whole functional system was manufactured with high performance by taking advantage of silicon-based MEMS fabrication technology. Then, a curved display was manufactured as a demonstration. The LED chips were picked up, placed, and soldered onto the fabricated silicon-based planar platform. Next, the customized conformal vacuum transfer printing (CVTP) technique was developed to transfer the system onto arbitrary curved surfaces for encapsulation. Finite element analysis was used to simulate the deformation of the proposed structures, and tensile experiments were conducted to verify the mechanical properties of the VSC-enabled system.

## Results

### Silicon-based MEMS fabrication for the planar configuration

**Fig. 2:**
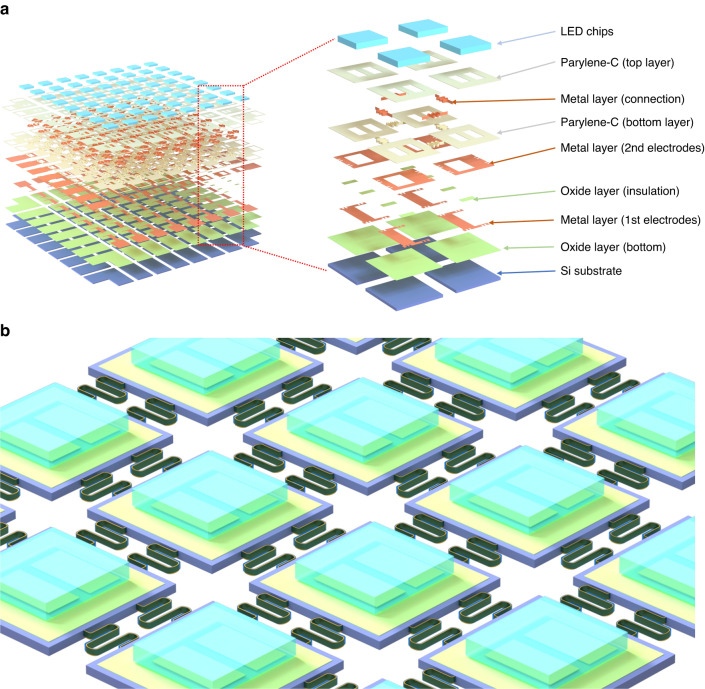
Design concept of the VSC-enabled stretchable electronic device. **a** The structure and material details for the multilayer of an 8 × 8 LED array. **b** Three-dimensional stretchable and curved display in a planar form

Stretchable and deformable functional island nodes interconnected with VSCs were manufactured on a planar Si wafer. The multilayer structure of an 8 × 8 LED array display device is illustrated in Fig. 2. Figure 2a presents details of the materials and patterns of different layers. At the start of the process, silicon islands were manufactured as vehicles to have functional layers and structures deposited and patterned. The fabrication steps here involve photolithography and etching to define the oxide layer as the mask from the backside of the silicon wafer. The LED array is controlled individually with the passive matrix configuration, consisting of three metal layers, two of which are used for the anodes and cathodes, with the third being for the electrical interconnection between the island nodes. Metal layers were deposited using a sputtering machine, and wet etching processes were followed to pattern the metal layers. In addition, an oxide layer was deposited as an insulating layer between metal layers. After deposition and defining electrode pads for bonding LED chips onto the silicon islands, grooves with a width of 10 μm and a depth of 40 to 50 μm, which mold the interconnects between island nodes, were patterned using the deep reactive ion etching system (DRIE). Three layers, including Parylene-C, metal, and Parylene-C, were deposited successively to fill the grooves. Initially, the first Parylene-C layer was deposited and patterned at the bottom; by adjusting the thickness of the Parylene-C layer, the metal layer can be deposited and positioned close to the neutral plane of the interconnects, thereby enhancing its mechanical robustness. Subsequently, the second Parylene-C layer was deposited to fill the groove and protect the inner metal layer. Photolithography and reactive-ion etching (RIE) were used here to define and pattern the Parylene-C and oxide layers. Finally, DRIE and RIE were both utilized sequentially to etch silicon from the backside of the Si wafer, release the VSCs in the grooves and obtain the interconnected silicon-based functional vehicles. Then, LED chips were picked and placed and bonded at electrode pads on each island node. Figure 2b shows a 3D schematic of silicon-based functional vehicles with LED chips applied. Furthermore, multiple interconnections were designed to connect island nodes. They worked as the redundancy for each other in the current design to improve the reliability of the device. They can also work as different data connections for future more complicated circuits. The detailed processes are described in the “Methods” section.

**Fig. 3:**
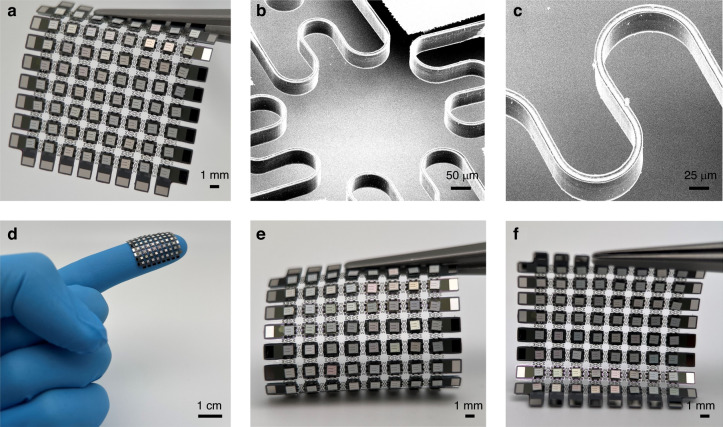
Images of the functional device. **a** A photograph of an 8 × 8 LED array with released VSCs hung by a tweezer. **b** SEM image of released VSCs between functional island nodes in (**a**). **c** A magnified image of the VSC structure in (**b**). **d** A photograph of the 8 × 8 LED array wrapped on a finger. **e**, **f** Photographs of the 8 × 8 LED array bent in different directions while maintaining structural integrity and electrical properties

Figure 3 shows optical and SEM images of the device after releasing interconnects and bonding BXDA 40 mil × 40 mil LED chips (Bridgelux) onto the functional islands. Low-temperature solder paste was manually dispensed using a three-axis motion platform. Then, a Manual Flip Chip Bonder (FINEPLACER 96 “Lambda”) was used to pick and place LED chips onto silicon islands, and the reflow process was followed to fix chips on the electrodes. A 22 mm × 22 mm display with an 8 × 8 LED array is presented in Fig. 3a. The SEM image of VSC is detailed in Fig. 3b, c, with a metal layer sandwiched between Parylene-C layers. The display can be wrapped onto a finger (Fig. 3d) and bent in different directions (Fig. 3e, f). The device shows high reliability during deformation and fits into different curved shapes while maintaining its structural integrity and electrical performance.

### Conformal vacuum transfer printing

**Fig. 4:**
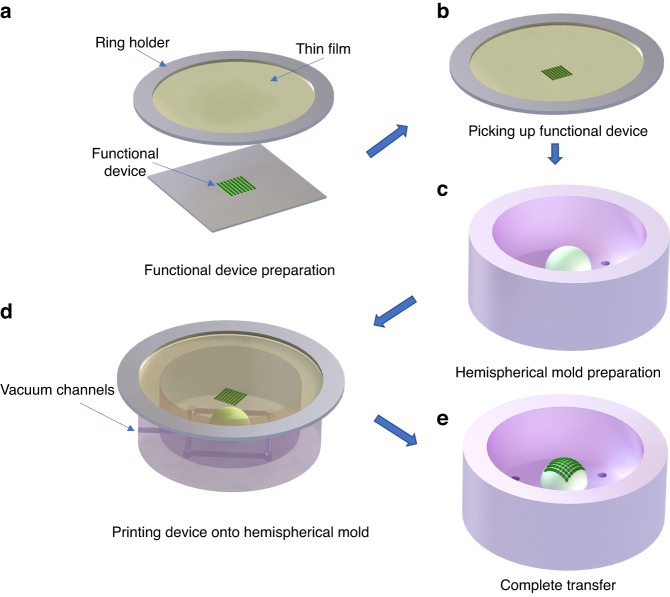
Schematic of conformal vacuum transfer printing (CVTP) processes. **a** Functional device preparation. **b** Picking up functional device. **c** Hemispherical mold preparation. **d** Printing device onto hemispherical mold. **e** Complete transfer

A conformal vacuum transfer printing (CVTP) method was proposed for functional device pick-and-place processes. Figure 4 shows the detailed process of CVTP to transfer printing a functional device onto the curved surface (video clip available in Supplementary Movie 1). The process began with the preparation of functional devices manufactured using silicon-based MEMS fabrication technology. First, the device was picked up from the planar substrate utilizing a prepared thin polymer film with the edge fixed by an acrylic ring. Then, the device was transferred onto an arbitrary curved surface in a customized vacuum chamber. During the picking-up process, the thin film was fastened to the ring holder, allowing for controlled movement up and down. The thin film was lowered gently until it completely contacted the device, enabling the device to be picked up from the substrate. The ring holder was designed with an inner diameter that matches the outer size of the customized chamber, ensuring that the device was aligned at the center of the curved surface during the printing process. Air channels were built into the bottom of the chamber to connect it with an external pump. Next, the printing process successively completed pumping and venting steps. During the pumping step, the edge of the thin film came into close contact with the chamber, causing the rest of the thin film to stretch uniformly and thus printing the device onto the prepared curved mold. When the device had entirely made contact with the curved surface, the chamber was vented, resulting in the device being retained on the curved substrate due to the treated surface utilizing uncured PDMS.

### VSC-enabled curved displays

The CVTP approach was used to transfer the 8 × 8 LED array onto different complex curved substrates, including hemispherical substrates, saddle-shaped substrates, and irregularly curved surfaces. Three different concave curved molds were designed and manufactured through the 3D printer, including a hemispherical mold with a 2.5 cm diameter, a saddle surface mold with a 2.5 cm diameter curvature at the saddle point, and an irregular curved surface with dimensions of 2.5 cm * 2.5 cm. Then, the corresponding convex curved substrates were fabricated using the demolding process. Additional details of these steps are provided in the “Methods” section.

**Fig. 5:**
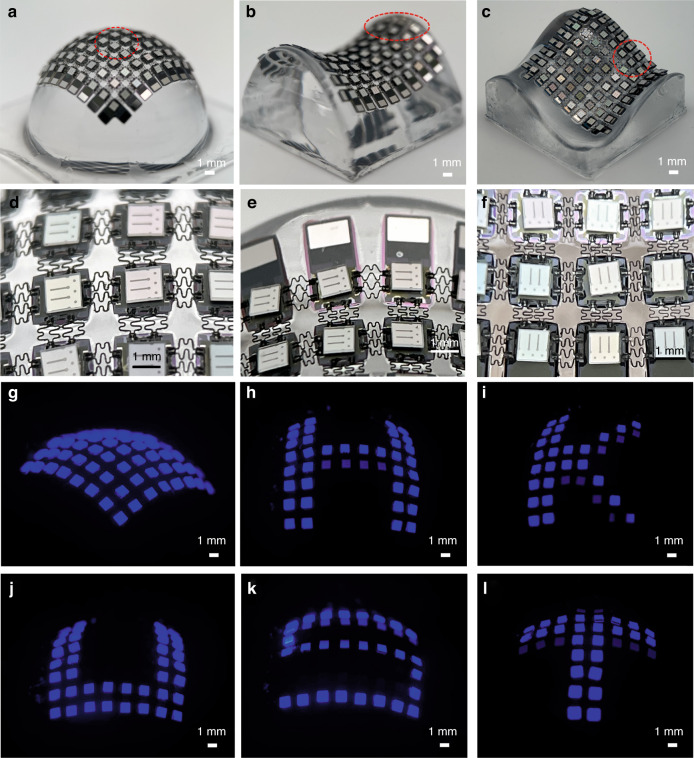
Images of curved displays. **a** Image of the 8 × 8 LED array packaged on a hemispherical mold. **b** Image of the 8 × 8 LED array packaged on a saddle-shaped substrate. **c** Image of the 8 × 8 LED array wrapped on an irregular curved substrate. **d** A partially magnified image of the LED array interconnected with VSCs in (**a**). **e** A partially magnified image in (**b**). **f** A partially magnified image in (**c**). **g** Image of all LEDs lit up on the curved substrate. **h**–**l** Images of the curved display presenting the letters “HKUST” individually

Figure 5 presents the VSC-enabled curved displays. A diagonal view of the hemispherical display is demonstrated in Fig. 5a. The LED array is wrapped intimately onto the curved surface, and a magnified image (Fig. 5d) reveals different deformations of the interconnects in the center region to better fit the curved surface. Interconnects in the middle area are in a stretched status, while interconnects in the surrounding parts show a state of compression. The saddle-shaped display (Fig. 5b,e) and the irregular curved display (Fig. 5c, f) are also presented to demonstrate the conformability of the VSC-enabled curved display and the feasibility of the CVTP technology. The LED arrays are configured with a passive matrix to drive each chip individually and display designed patterns. Figure 5g presents all LED pixels on a hemispherical surface lighting up, and Fig. 5h to Fig. 5l demonstrate the letters “HKUST” individually (corresponding video clip shown in Supplementary Movie 2).

### Mechanical properties

**Fig. 6:**
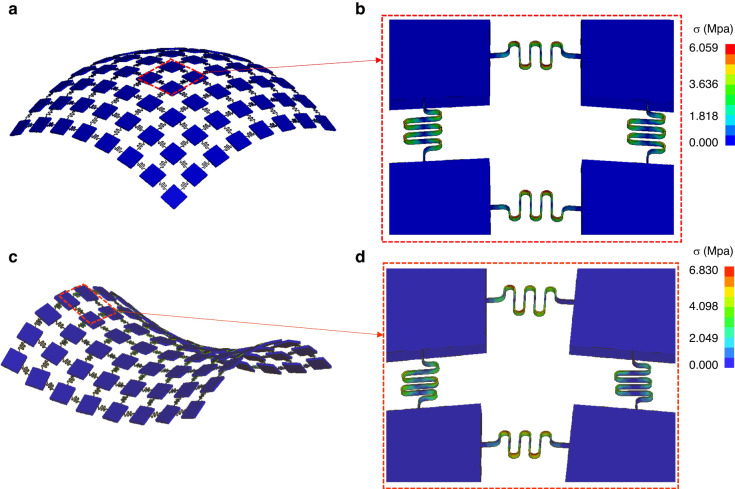
FEA results for 8 × 8 island nodes interconnected with VSCs covered intimately onto curved surfaces. **a** An overall view of the stress distribution for the curved device covering the hemispherical surface. **b** A magnified picture of the stress distribution in the middle region in (**a**). **c** An overall view of the stress distribution for the curved device covered on the saddle surface. **d** A magnified picture of the stress distribution in the marginal region in (**c**)

To further investigate the mechanical properties of the VSC-enabled functional device after transfer printing onto curved surfaces, FEA simulation was conducted to analyze representative 8×8 island nodes interconnected with VSCs covered intimately onto curved surfaces, including hemispherical surfaces and saddle surfaces. The geometric dimensions here are identical to those of the curved electronics presented in Fig. 5. Figure 6 shows the simulation results, including an overall view of the stress distribution (Fig. 6a, c) and magnified pictures of the stress distribution. For the device covered on the hemispherical surface, the maximum stress occurred at the vertical serpentine conductors in the middle region, with a value of 6.1 Mpa (marked in a red box, as shown in Fig. 6b). For the device covered on the saddle surface, the maximum stress occurred in the marginal region, with a value of 6.8 Mpa (marked in a red box, as shown in Fig. 6d). Both of the maximum stresses are far lower than the tensile strength of copper at approximately 210 Mpa. Therefore, VSC-enabled stretchable and curved electronics effectively relieve stress concentrations and greatly enhance the conformability and reliability of curved electronics.

The LED arrays demonstrated the ability to be stretched, bent, folded, and wrapped intimately onto curved surfaces. However, deformation also occurs during the CVTP process. To investigate the stretchability and reliability of the device, tensile experiments were performed for vertical serpentine conductors and the VSC-enabled LED array. Details of these experiments are provided in Supplementary Note 5. The VSC can be stretched up to 350% while maintaining mechanical integrity, and the electrical resistance is highly stable, only changing less than 2% under a 300% applied strain (Fig. S5). In addition, the VSC-enabled LED array shows favorable reliability after 100 cycles at 100% expansion in the durability test, and the corresponding video clip is available in Supplementary Movie 4. Redundant interconnects were designed and incorporated to interconnect island nodes and further improve the reliability of the device. The stretchability can be further increased through different designs of the VSC interconnect and optimization of the ratio of the island nodes and the spacing. These related improvements will be investigated in future research.

## Discussion

To summarize, a VSC interconnected functional platform was proposed and manufactured to create three-dimensional stretchable and curved electronics. This strategy provides a new approach for fabricating stretchable and curved electronics. Compared with the in-plane serpentine structure, VSCs can avoid sharp stress concentrations at the arc region during deformation and effectively improve their mechanical and electrical properties. By taking advantage of silicon-based MEMS fabrication technology, stretchable and curved platforms can be constructed and integrated with multifunctional devices. In addition, CVTP technology was developed to transform manufactured devices into arbitrarily curved surfaces. The excellent deformability of VSCs enabled the elastomeric film to pick up the device and intimately contact complicated curved surfaces during the transfer printing process. An LED array with a passive matrix configuration was manufactured and packaged onto hemispherical and saddle surfaces to demonstrate the feasibility of the approach. The mechanical properties were investigated numerically and experimentally. The VSC-enabled devices possess high stretchability, stability and reliability, making them promising candidates for stretchable and curved electronics.

## Methods

### Silicon-based microfabrication

**Fig. 7:**
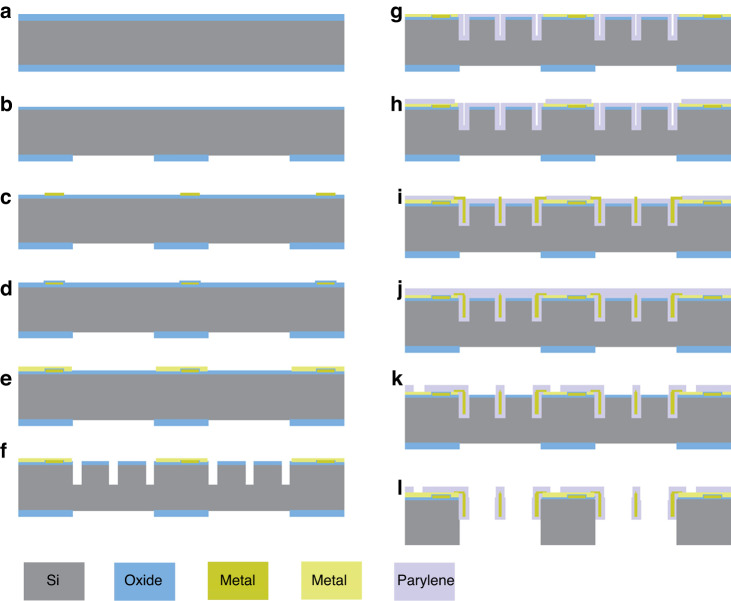
Silicon-based microfabrication process for stretchable and curved electronics. **a**, **b** Pattern on the reverse side of the silicon wafer. **c**–**e** Two metal layer deposition and patterning for electrodes on islands. **f** Groove patterning and etching using the DRIE system. **g**–**j** Materials deposition to fill in the groove and pattern the Parylene-C layers and metal layer to form a sandwich structure for interconnects between islands. **k** Patterning and etching the top layer of Parylene-C. **l** Dry etch the silicon substrate from the reverse side using DRIE and RIE systems to release the conductors and obtain a free standing system

The fabrication processes of VSC-enabled stretchable and curved electronic devices are detailed in the following section (Fig. 7).

The VSC interconnected functional device was prepared from double-side polished silicon wafers with 3-μm-thick thermal oxide on top. The fabrication of the device started with photolithography to pattern the reverse-side oxide layer, which was designed as the mask layer in the following etching steps. The photolithography process included deposition of the adhesion promoter hexamethyldisilazane (HMDS), spin-coating of photoresist (HPR 506) at 4000 rpm for 1 min and soft baking on a hotplate at 110 °C for 2 min, then ultraviolet exposure (λ = 365 µm) with a Karl Suss MA6 aligner (25 mW/cm^2^) for 9.5 s, followed by development with FHD-5 for 60 s, and hard baking in an oven at 120 °C for 30 min. Thereafter, the reverse-side silicon oxide layer was etched using Dry AOE Etcher by a mixture of gases C_4_F_8_ with a gas flow of 100 sccm for 12 min. Then, O_2_ Plasma was used to remove the photoresist.

The second step included deposition and patterning of the bottom electrode metal layer onto the front side of the Si wafer. The deposition was performed with a CVC-601 Sputter (SPT-CVC) for the first electrode metal layer (TiW/Cu, 30/500 nm) at a high sputtering rate (17.5 nm/min for TiW and 100 nm/min for Cu). Then, the metal layer was patterned by a photolithography process with 2-μm-thick HPR 506 photoresist, and the wet etching process used a copper etchant and hydrogen peroxide at 60–70 °C.

The third step included deposition of a thin silicon dioxide layer, which functioned as an insulating layer between two electrode metal layers. A 100-nm-thick oxide layer was formed using STS PECVD with a high deposition rate (55 nm/min) at 300 °C. After that, the deposition and patterning of the second electrode metal layer (TiW/Cu, 30/500 nm) were performed on the top of the insulating layer.

The fourth step included patterning and dry etching of the three-dimensional deep trench for interconnects. The 3-μm-thick HPR 506 photoresist was spin-coated onto the front side of the wafer, followed by UV exposure, developing, hard baking, and descum. Thereafter, the oxide layer on the surface was first etched using 777 etchant (purchased from FUJIFILM Electronic Materials U.S.A., Inc.), and then approximately 50 μm-depth grooves were dry-etched using the DRIE etcher system. During the DRIE etching process, alternative passivation and etching processes were employed to obtain a highly anisotropic deep etch with a low undercut.

Thereafter, the first layer of Parylene-C was coated on the front side of the silicon wafer using the SCS Labcoter 2 (PDS2010) vacuum deposition system. The surface of the silicon wafer was treated with a mixture of DI water:IPA:A174 at a ratio of 100:100:1 (by volume) to promote adhesion between the silicon wafer and the Parylene C layer. Then, the silicon wafer was placed on a rotating platform during the process to achieve a uniform thin film. After, the 8-µm-thick photoresist was spray-coated on the surface of the Parylene-C layer to serve as a masking layer for opening the metal layers underneath. The photoresist used here was a mixture of AZ9260:MEK:PGMEA with a ratio of 1:8:1 (by weight), which was customized for the EVG Spray coating machine. After exposure and development, the RIE system was employed to dry etch the Parylene-C layer with an etching rate of 0.5 μm per minute.

Then, another metal layer was deposited to interconnect the island nodes, followed by the deposition and patterning of a second layer of Parylene-C on the front side to encapsulate and protect the internal functional layers. The last step was to dry etch the silicon wafer from the backside, and the patterned oxide layer was used as a mask layer during the etching process. Both the DRIE and RIE systems were employed for VSC interconnected island node release and substrate thinning.

The Manual Flip Chip Bonder (FINEPLACER 96 “Lambda”) and reflow hotplate were employed during the LED chip (30 mil*30 mil) pick-and-place packaging process. A low-temperature solder paste was used here to ensure the mechanical properties of Parylene-C.

### Supplementary information

Supplemental materials

The conformal vacuum transfer printing (CVTP) process

Sphere display

Saddle display

Repeatability test
