# Supplementary material for: Vertical serpentine interconnect-enabled stretchable and curved electronics
Source: Microsyst Nanoeng. 2023 Nov 27;9:149. doi: 10.1038/s41378-023-00625-w (PMC10679150; doi:10.1038/s41378-023-00625-w)
Supplement: Supplementary file 1 — Supplemental materials [file 41378_2023_625_MOESM1_ESM.docx]

## Supplementary materials

**Vertical serpentine interconnects enabled stretchable and curved electronics**

*Rui Jiao^1#^, Ruoqin Wang^1#^, Yixin Wang^1^, Yik Kin Cheung^1^, Xingru Chen^1^, Xiaoyi Wang^1^, Yang Deng^1^*, Hongyu Yu^1,2,^**

^1^ Department of Mechanical and Aerospace Engineering, The Hong Kong University of Science and Technology, Kowloon, Hong Kong SAR 999077, China

^2^ HKUST Shenzhen-Hong Kong Collaborative Innovation Research Institute, Shenzhen, Guangdong 518045, China

^#^ The authors contribute equally.

Email: [hongyuyu@ust.hk](mailto:hongyuyu@ust.hk)

## Supplementary Note 1: Parylene-C layer preparation

Parylene-C (Galentis) layer was coated using the SCS Labcoter 2 (PDS 2010) vacuum deposition system. The sample was placed on a rotating platform during the process to ensure the uniform coating film was obtained. 10 gram of dimer was used here to obtain a thin coating layer with a thickness of approximately 7.5μm.

## Supplementary Note 2: Thin polymer film preparation

The thin polymer film was prepared by Ecoflex (Smooth-On Ecoflex 00-30). A and B were first mixed. Then, the liquid precursor was spin-coated onto a silicon wafer at 500rpm, and hardbaked at 75℃ for about 2hrs. The thin film was peeled off from the wafer using a laser-cut acrylic ring.

## Supplementary Note 3: Curved substrates preparation

The curved substrates were prepared using a customized mold. The mold was 3D printed, and the surface was coated with a thin layer of Parylene-C. Liquid silicone PDMS (Sylgard 184, a ratio of monomer to cross-linker is 10:1) was poured into the treated mold. The mold was then baked at 75℃ for about 2hrs in the oven.

## Supplementary Note 4: Finite element analysis (FEA)

The finite element analysis was conducted to simulate the representative 8×8 island nodes interconnected with VSCs covered intimately onto curved surfaces, including the hemispherical surface and saddle surface. The geometric dimensions of the finite element models are identical to the curved electronics presented in manuscript. Tetrahedral elements are used to discretize the geometry of silicon island nodes and curved substrate including the hemispherical substrate and the saddle substrate. Composite shell element is used for the vertical serpentine conductors between the island nodes. Linear elastic model is used to demonstrate the material behavior for the curved device. For the silicon island nodes, the elastic modulus (E) and Poisson’s ration (μ) are $E_{Si}$=130 GPa, $\mu_{Si}$= 0.27. For the vertical serpentine conductors, they are composed of Parylene C/Cu/Parylene C (5 μm/0.5μm/ 5μm), where $E_{Cu}$=119 GPa and $\mu_{Cu}$= 0.34 for Cu, and $E_{Pary}$=2.76 GPa and $\mu_{Pary}$= 0.4 for Parylene C. For the curved substrate fabricated using PDMS, $E_{PDMS}$=0.8 GPa and $\mu_{PDMS}$= 0.5.


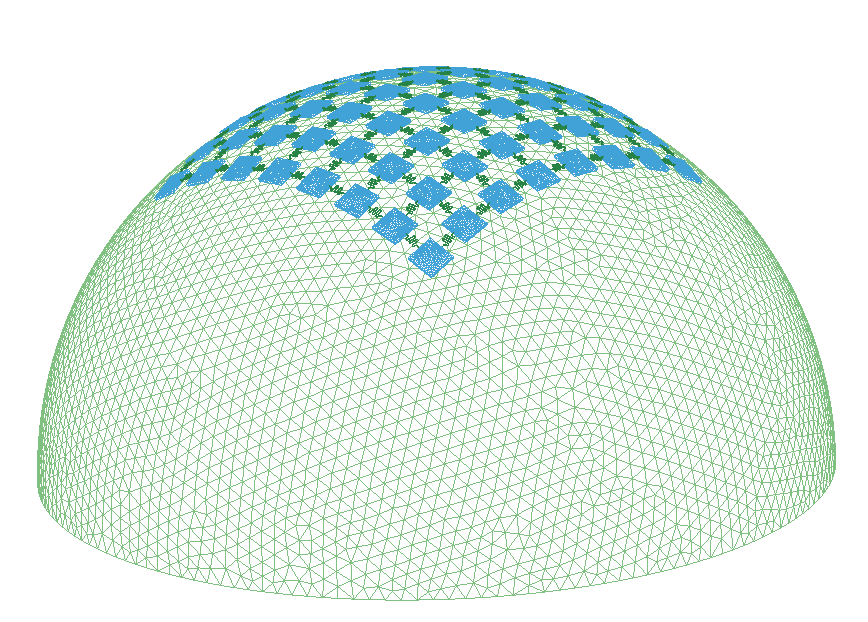


**Fig. S1 The full finite element model with discretized elements for the hemispherical shaped device.**


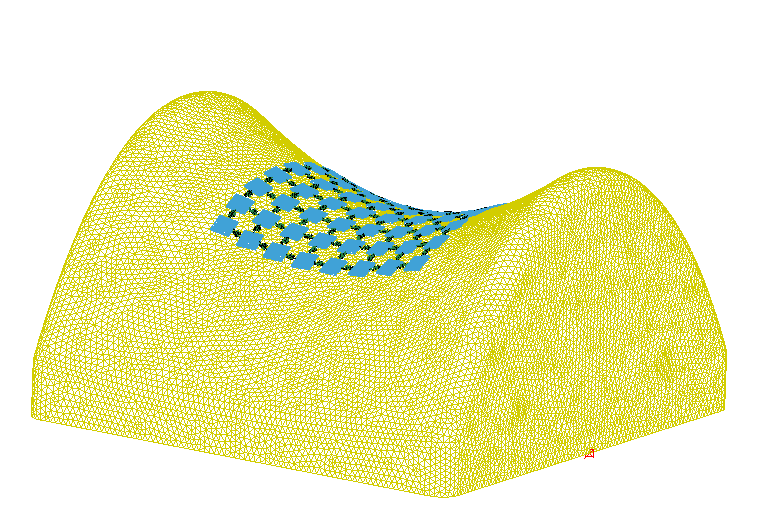


**Fig. S2 The full finite element model with discretized elements for the saddle shaped device.**

## Supplementary Note 5: Mechanical experiments

In order to better understand the mechanical and electrical performance of the VSC during deformation, the vertical serpentine conductors were designed and fabricated with the same critical parameters, with a radius of 60μm, and height of 50μm. The length of the test conductors was designed to be 0.5mm, and a distance of 5mm to better conduct the experiment. Fig. S3 shows the vertical serpentine conductor defined by key parameters, and the stretchability of the conductor is mainly determined by the radius, the length of the arm, and the height.


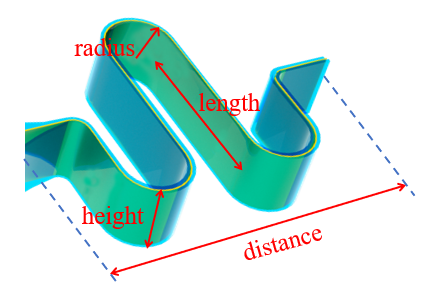


**Fig. S3 Vertical serpentine conductor defined by key geometric parameters.**

Fig. S4 presents a series of images for the vertical serpentine conductor under different stretching status. The vertical serpentine conductor can be stretched up to 350% while maintaining its mechanical integrity and only has in-plane deformation.


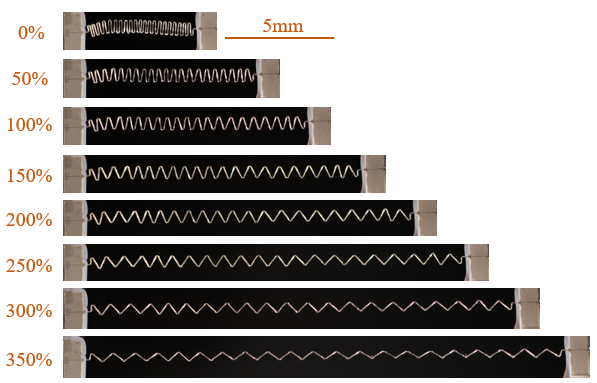


**Fig. S4 A series of images for the vertical serpentine conductor under different stretching status from 0 to 350%.**

Fig. S5 presents the relative resistance changes of the vertical serpentine conductor. The electrical resistance only has slight change when being stretched up to 300%, showing super stretchability and stability of the vertical serpentine conductors.


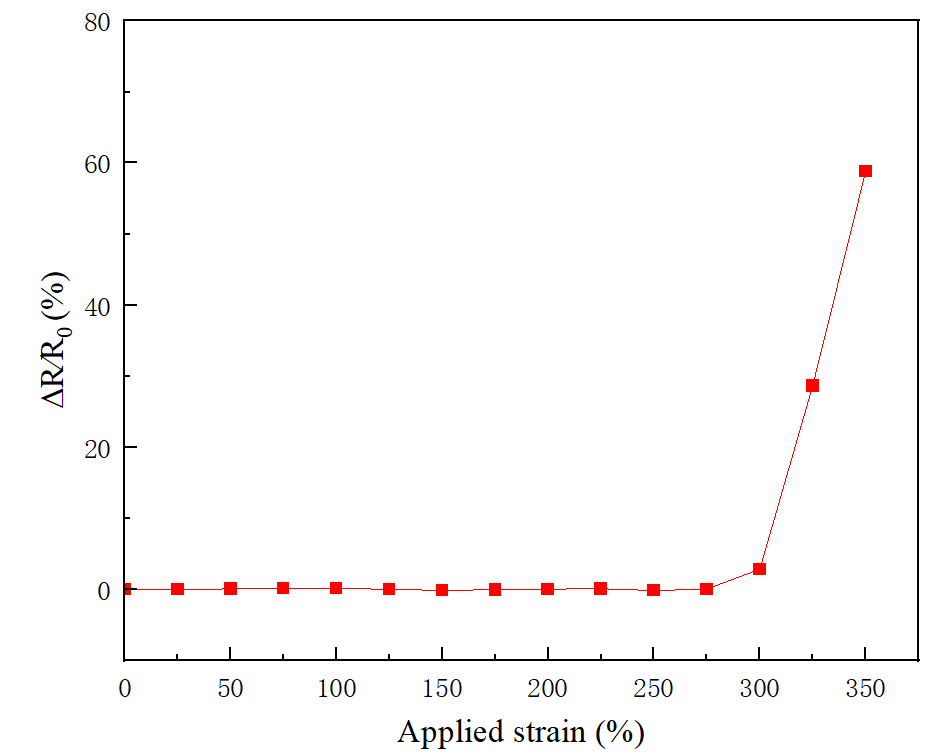


**Fig. S5 The relative resistance changes of the vertical serpentine conductor under a gradually increasing applied strain from 0 to 350% at 25% strain intervals.**

A biaxial moving platform was also designed to stretch the LED array in both X and Y directions. The device was packaged on a thin film prepared by Ecoflex (Smooth-On Ecoflex 00-30) as a substrate. The island nodes were semi-embedded in the thin film substrate while the VSC interconnects were suspended on the surface of the film. The substrate enables the device to be stretched uniformly without affecting the stretchability of VSC interconnects. The initial size of the 5×5 LED array was 10mm×10mm with an interconnect length of 0.5mm (Fig. S6a). After biaxial stretching, the size became 12mm×12mm. The length of an interconnect was 1mm (Fig. S6b).


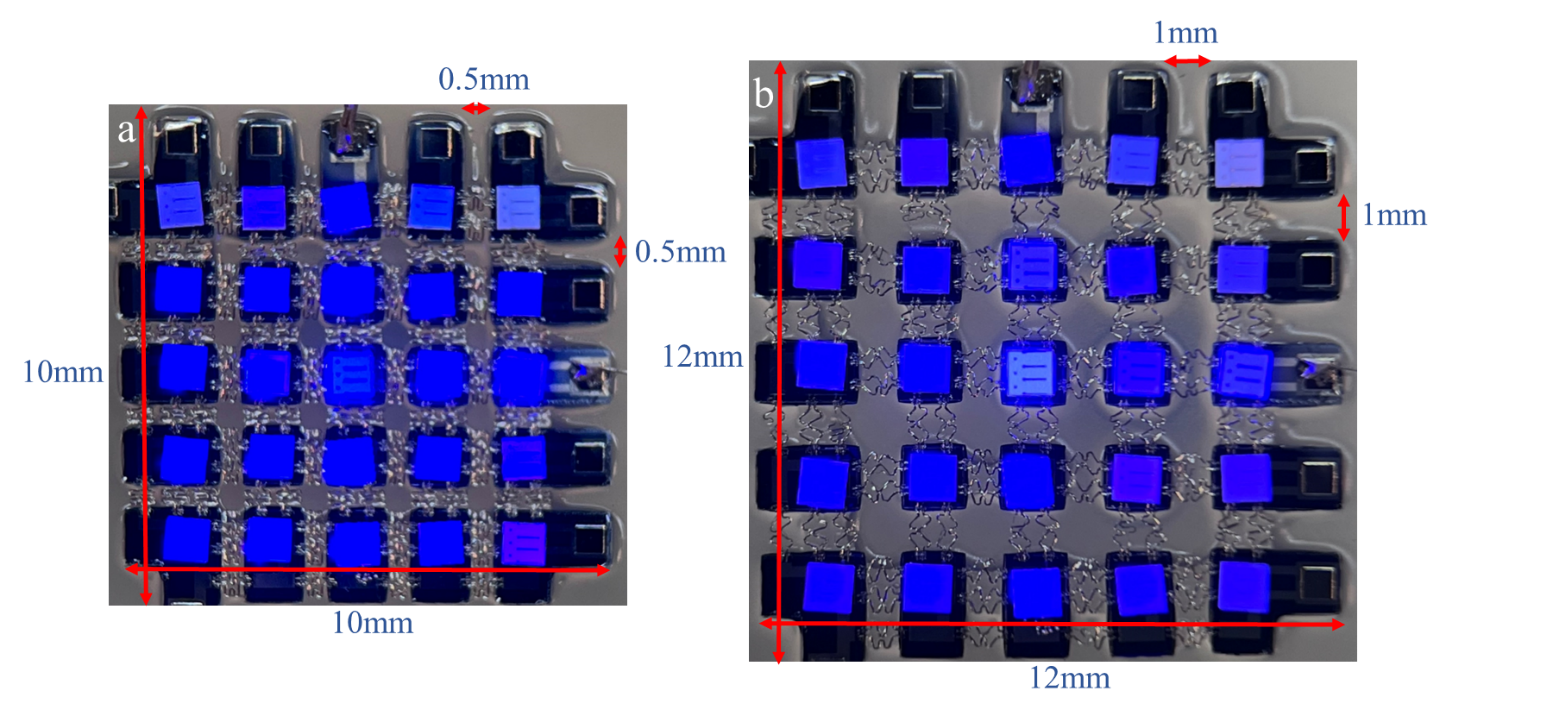


**Fig. S6 Images of the biaxial tensile experiment. a** The original status of a 5×5 LED array on the stretchable substrate, with all LEDs lit up. **b** The stretching status of the 5×5 LED array, with all LEDs lit up.

## Supplementary movies

Movie 1: The whole process to transfer printing the functional device onto the curved surface utilizing the conformal vacuum transfer printing (CVTP) technology.

Movie 2: Dynamically presenting the letters “HKUST” individually on the hemispherical surface.

Movie 3: Dynamically presenting the letters “HKUST” individually on the saddle-shaped surface.

Movie 4: Repeatability test for the 5×5 LED array under 100% expansion.
